# A single-center retrospective study of short-term efficacy and safety of blinatumomab in the treatment of high-risk acute lymphoblastic leukemia

**DOI:** 10.1186/s12885-026-16389-1

**Published:** 2026-06-22

**Authors:** Yixuan Ma, Luyang Chang, Lin Wang, Lin Chen, Xudong Wei, Ruihua Mi

**Affiliations:** 1 The Affiliated Cancer Hospital of Zhengzhou University & Henan Cancer Hospital, Zhengzhou, 450008 China; 2 Department of Biochemistry and Molecular Biology, School of Basic Medical Sciences, Zhengzhou University, Zhengzhou, 450001 China

**Keywords:** Acute lymphoblastic leukemia, Blinatumomab, Minimal residue disease, Relapse

## Abstract

**Background:**

Treatment outcomes for high-risk B-ALL remain suboptimal. Blinatumomab, a CD19/CD3 bispecific T-cell engager, has shown efficacy in trials, but real-world data in Chinese high-risk patients are limited.

**Methods:**

This single-center retrospective study analyzed 29 high-risk B-ALL patients treated with blinatumomab (7–28 days/cycle). We assessed short-term efficacy (MRD negativity rate), safety, and the impact of genetic factors and treatment duration.

**Results:**

The overall MRD negativity rate after one cycle of blinatumomab was 82.8% (24/29). All patients with the Ph-like subtype (3/3, 100%) and 71.4% (10/14) of Ph+ patients achieved MRD negativity. However, clinical outcomes were inferior in patients harboring specific genetic alterations: 50% (2/4) of patients with the *BCR::ABL1* T315I mutation experienced early relapse, and only one of two patients with *E2A::PBX1* rearrangement achieved transient MRD negativity. The median event-free survival (EFS) was 9.6 months (95% CI: 6–16.75). Grade ≥ 3 hematologic toxicity occurred in 48.3% (14/29) of the patient cohort.

**Conclusion:**

Blinatumomab achieved high short-term MRD response rates in high-risk B-ALL. Inferior outcomes appeared to be associated with the presence of *BCR::ABL1* T315I mutations and *E2A::PBX1* rearrangements.

**Supplementary Information:**

The online version contains supplementary material available at https://doi.org/10.1186/s12885-026-16389-1.

## Background

B-cell acute lymphoblastic leukemia (B-ALL) is a hematologic malignancy characterized by the aberrant proliferation of immature B lymphocytes, which suppresses normal hematopoiesis in the bone marrow and peripheral blood. It is the most common subtype of adult ALL, accounting for approximately 70–80% of cases [1, 2]. In recent years, treatment outcomes for adult B-ALL have improved significantly, benefiting from the optimization of pediatric-inspired multi-agent chemotherapy regimens and the application of novel molecularly targeted drugs. This has led to complete remission (CR) rates of 80%-90% with first-line induction therapy and a 5-year overall survival (OS) of approximately 50–60% [1]. However, high-risk adult B-ALL is characterized by high heterogeneity and relapse rates, with a 5-year OS of only about 40% [2]. Its definition relies not only on traditional clinical indicators (such as age, white blood cell count, treatment response) but is also closely associated with molecular genetic abnormalities (e.g., Tp53 mutations, Ph-like subtype) and minimal residual disease (MRD) dynamics, which are independent risk factors for predicting relapse [3, 4]. Even with intensive chemotherapy or allogeneic hematopoietic stem cell transplantation (allo-HSCT), the risk of relapse remains significant for these patients [2].

Breakthroughs in immunotherapy have provided a new dimension for treating high-risk ALL. Blinatumomab is a bispecific T-cell engager (BiTE) that targets both CD19 on B-cells and CD3 on T-cells. It specifically binds to CD19-positive B cells and the CD3 molecule on T cells, bringing them into direct contact and thereby activating the T cells’ cytotoxic potential against cancer cells [5–7]. Furthermore, blinatumomab can trigger intrinsic T-cell signaling, leading to the expression of key activation markers such as CD69 and CD25, upregulation of the cell adhesion molecule CD2, and transient release of inflammatory cytokines. This cascade of events results in comprehensive T-cell activation and proliferation, ultimately destroying tumor cells [7]. The TOWER phase III clinical trial demonstrated that in adults with relapsed/refractory ALL, blinatumomab compared to standard chemotherapy achieved a median OS extension from 4.0 months to 7.7 months and increased the MRD negativity rate to 76% (vs. 48%), leading to its approval as the first BiTE for ALL [6]. For specific high-risk populations, the French Graall QUEST study confirmed that blinatumomab compared to chemotherapy increased the MRD negativity rate by 19%, reduced the cumulative incidence of relapse by 59%, and the risk of events by 51%, showing significant benefit particularly for patients with IKZF1 deletion [8]. However, data on the efficacy of blinatumomab for treating high-risk B-ALL in the Chinese population remain scarce.

Based on single-center retrospective data, this study systematically analyzes the short-term efficacy of blinatumomab in high-risk ALL patients. The study cohort includes patients with high-risk features such as PH+/Ph-like subtype, abnormal chromosomal karyotype, and MRD positivity. It aims to evaluate the short-term efficacy (e.g., MRD negativity rate) of blinatumomab and its relationship with genetic abnormalities, as well as the molecular markers associated with resistance. By integrating molecular subtyping and dynamic MRD monitoring data, this research aims to provide evidence for the immunotherapy of high-risk ALL patients and reveal optimal application strategies for blinatumomab in complex clinical scenarios.

## Materials and methods

### Subjects 

A retrospective analysis was conducted on data from high-risk B-ALL patients treated with blinatumomab at the Affiliated Cancer Hospital of Zhengzhou University between September 2021 and October 2024. Diagnosis and classification were based on the WHO 2022 (5th edition) criteria integrating cytomorphology, immunology, cytogenetics, and molecular biology (MICM). Risk stratification was defined according to the Chinese Guidelines for Diagnosis and Treatment of Adult Acute Lymphoblastic Leukemia (2024 edition) [4, 9]. This study was approved by the Ethics Committee of The Affiliated Cancer Hospital of Zhengzhou University. Informed consent was obtained from all individual participants or their legal guardians included in the study.

### Treatment methods

Blinatumomab was administered via continuous intravenous infusion over 24 h. The planned treatment duration per cycle was 28 days; however, the actual duration ranged from 7 to 28 days due to variations in individual treatment response and economic factors. In this study, patients with high-risk B-ALL were treated with blinatumomab regardless of their baseline MRD status. For patients who have achieved MRD negativity, treatment indications include: (1) consolidation therapy to eradicate potential residual leukemia clones undetected by routine MRD testing, particularly in patients with high-risk genetic features (such as *BCR::ABL1, IKZF1* deletion, or complex karyotypes); (2) prophylactic therapy to reduce the risk of molecular relapse; or (3) as a bridging therapy prior to allo-HSCT. Therefore, the scope of treatment is not limited to MRD-positive patients. For patients with *BCR::ABL1* gene mutations, blinatumomab was combined with a Tyrosine Kinase Inhibitor (TKI). Supportive care during myelosuppressive phase included: transfusion of suspended red blood cells when hemoglobin concentration fell below 60 g/L or clinical symptoms appeared; transfusion of apheresis platelets when platelet count was < 20 × 10⁹/L or there was a clinical bleeding tendency; and routine administration of G-CSF when the absolute neutrophil count was < 0.5 × 10⁹/L, continued until the neutrophil count recovered to > 0.5 × 10⁹/L.

### Efficacy evaluation 

The efficacy of ALL treatment was assessed following the criteria outlined in the Chinese Guidelines for Diagnosis and Treatment of Adult Acute Lymphoblastic Leukemia (2024 edition) [10].

### Safety evaluation 

Hematological and non-hematological adverse events associated with blinatumomab were graded according to the CTCAE version 6.0 criteria. Cytokine Release Syndrome (CRS) was graded using the Lee criteria [11].

### Definitions 

Complete Remission (CR): Defined as the disappearance of all signs and symptoms of leukemia, absence of leukemic cells in peripheral blood differential, bone marrow blasts < 5%, no evidence of extramedullary leukemia, with neutrophil count ≥ 1.0 × 10⁹/L and platelet count ≥ 100 × 10⁹/L. CR with Incomplete Hematologic Recovery (CRi): Meets all CR criteria and is transfusion-independent, but with persistent cytopenia (neutrophil count < 1.0 × 10⁹/L or platelet count < 100 × 10⁹/L). Morphologic Leukemia-Free State (MLFS): A bone marrow blast count of less than 5% (by morphologic assessment), and the absence of blasts with Auer rods in the peripheral blood, and the absence of extramedullary leukemia. Partial Remission (PR): Post-chemotherapy reduction of bone marrow blast percentage by at least 50%, resulting in a level between 5% and 25%, accompanied by normalized peripheral blood counts. No Remission (NR): Failure to achieve either CR or PR after induction therapy. Refractory: Failure to achieve CR after ≥ 2 cycles of standard chemotherapy regimens. Relapse: Reappearance of leukemic cells in the peripheral blood, or bone marrow blasts ≥ 5%, or evidence of extramedullary leukemic infiltration in patients who had previously achieved CR.

### MRD monitoring and post-treatment follow-up 

MRD was dynamically monitored using bone marrow aspirate samples collected at baseline and post-treatment intervals. Two parallel methodologies were used for MRD assessment based on patient risk profiles: multiparameter flow cytometry (FCM-MRD) and quantitative polymerase chain reaction (PCR-MRD). FCM-MRD was utilized to detect abnormal leukemia-associated immunophenotypes, with MRD-negative status defined as baseline levels falling below the lower limit of detection (0.01%). For patients carrying specific molecular markers, PCR-MRD was performed to quantitatively evaluate the transcript levels of relevant fusion genes, including *BCR::ABL1* and *E2A::PBX1*. Follow-up was conducted via telephone, outpatient visits, and the hospital’s medical record registration system. The follow-up period ended in October 2024. Overall Survival (OS) was defined as the time from the start of blinatumomab treatment until the end of the follow-up period or the date of death for deceased patients. Event-Free Survival (EFS) was defined as the time from the start of blinatumomab treatment to the first occurrence of disease relapse, disease progression, or death from any cause. Patients who remained alive without an event were censored at the date of last follow-up.

### Statistical analysis 

Statistical analyses were performed using SPSS software (version 27.0). Continuous variables following a normal distribution were described using Mean ± Standard Deviation (SD), while non-normally distributed data were described using the Median (Interquartile Range, IQR) or median. Categorical variables were described using frequency (percentage). Survival outcomes (EFS, OS) were estimated using the Kaplan–Meier method, and median survival times are reported with 95% confidence intervals (CIs) where applicable. Given the small sample size (N = 29) and the limited number of samples within specific genetic subgroups, to avoid overinterpreting analyses with insufficient statistical power, this study largely refrained from conducting formal hypothesis testing (e.g., calculating p-values for subgroup comparisons) and instead employed descriptive statistical methods to explore potential trends. Due to the limited sample size and the risk of overfitting, no multivariate regression models were constructed. All reported differences should be considered exploratory findings, and the relevant results require validation in larger, independent cohorts.

## Results

### Patient baseline characteristics 

From September 2021 to October 2024, 29 high-risk ALL patients were enrolled, comprising 16 males (55.2%) and 13 females (44.8%), with a median age of 48 years (range: 15–68). Details are shown in Table 1 and Supplementary Table 1. Genetic abnormalities included: BCR::ABL1 fusion gene positivity in 14 cases (48.3%; including 5 cases of BCR::ABL1 P190 and 9 cases of *BCR::ABL1* P210, with 4 cases harboring concomitant *BCR::ABL1* T315I mutations), *E2A::PBX1* rearrangement in 2 cases (6.9%), *TP53/DNMT3A* mutations in 2 cases (6.9%), *FLT3-TKD* mutation in 1 case (3.4%), and *IKZF1* deletion/mutation in 6 cases (20.7%). Cytogenetic abnormalities: complex karyotype in 10 cases (34.5%) and t(9;22) in 9 cases (31.0%). Pre-treatment MRD levels: The median flow cytometry MRD was 0.18% (range: 0–92.16%), with 13 patients (44.8%) having MRD ≥ 0.1%. The median bone marrow blast percentage was 0.07% (range: 0–94.9%). Among the 29 patients, 5 received a 28-day blinatumomab course, 3 received 21 days, 12 received 17 days, 2 received 14 days, 2 received 12 days, and 5 received 7 days. All 11 patients with baseline minimal residual disease (MRD)-negative status (defined as MRD levels below the limit of detection by flow cytometry) were high-risk ALL patients. Among them, 8 (72.7%) carried high-risk genetic abnormalities, including *BCR::ABL1* (*n* = 7), *IKZF1* deletion (n = 3), and complex karyotypes (*n* = 5). Blinatumomab was used for consolidation therapy in these patients.

**Table 1:** Baseline characteristics of high-risk ALL patients

Characteristic	Patients (*N* = 29)
Sex, n (%)	
Male	16 (55.17%)
Female	13 (44.83%)
Median Age, years	48 (15–69)
Age Group, years, n(%)	
15 ~ < 35	8 (27.59%)
35 ~ < 55	10 (34.48%)
55 ~ < 65	8 (27.59%)
≥65	3 (10.34%)
Molecular Genetics, n (%)	
* t(9;22)/BCR-ABL1+*	14 (48.28%)
* E2A-PBX1*Rearrangement	2 (6.90%)
* TP53/DNMT3A* Mutation	2 (6.90%)
Others	6 (20.69%)
None Detected	3 (10.34%)
Karyotype	
Normal	16 (55.17%)
1 ~ 4 Chromosomal Abnormalities	7 (24.14%)
Complex Karyotype (≥ 5 Abnormalities)	6 (20.69%)
Pre-treatment Status, n (%)	
Newly Diagnosed	1 (3.45%)
CR with FCM-MRD negative/PCR-MRD negative	4 (13.79%)
PR	1 (3.45%)
CR with FCM-MRD negative/PCR-MRD positive	6 (20.69%)
CR with FCM-MRD positive/ PCR-MRD positive	9 (31.03%)
Relapsed	8 (27.59%)
Baseline FCM-MRD, n (%)	
≥10^− 1^ to < 1(≥10% to < 100%)	6 (20.69%)
≥10^− 2^ to < 10^− 1^(≥1% to < 10%)	4 (13.79%)
≥10^− 3^ to < 10^− 2^(≥0.1% to < 1%)	3 (10.34%)
≥10^− 4^ to < 10^− 3^(≥0.01% to < 0.1%)	5 (17.24%)
Below the Lower Limit of Detection	11 (37.93%)

### Efficacy evaluation 

All patients were evaluable for efficacy. All patients with Ph-like subtype achieved MRD negativity (100%, 3/3). Among Ph+ patients, the MRD negativity rate was 71.4% (10/14). Among patients with *BCR::ABL1* T315I mutations, 3 achieved MRD negativity. Of the 2 patients with *E2A::PBX1* rearrangement, one achieved short-term MRD negativity but relapsed rapidly, while the other showed a rapid increase in fusion gene levels. One newly diagnosed patient achieved CR after blinatumomab treatment. Among 7 patients with morphological or extramedullary relapse, 6 achieved complete remission, and 5 achieved MRD negativity. Detailed efficacy evaluation information for the patients is presented in Table 2.

**Table 2:** Efficacy characteristics in high-risk patients

Parameter	MRD Negativity Rate
Overall MRD Negativity Rate	82.76%(24/29)
Molecular Genetics(By PCR-MRD)	
* BCR::ABL1*	71.43%(10/14)
No Additional Mutation	77.78%(7/9)
* T315I* Mutation	50.00%(2/4)
* V299L*,* F317L* Mutation	100%(1/1)
* E2A::PBX1*	50.00%(1/2)
* Ph-like*	100%(3/3)
* TP53*,* DNMT3A*	100%(2/2)
* EP300::ZNF384*	100%(2/2)
Other	100%(3/3)
None Detected	100%(3/3)
Karyotype	
Normal	81.25%(13/16)
1 ~ 4 Chromosomal Abnormalities	71.43%(5/7)
Complex Karyotype (≥ 5 Abnormalities)	100%(6/6)
Sex	
Male	75.00%(12/16)
Female	91.31%(12/13)
Age Group (Years)	
15 ~ < 35	55.00%(6/8)
35 ~ < 55	80.00%(8/10)
55 ~ < 65	87.50%(7/8)
≥65	100%(3/3)
Treatment Duration (Days)	
7	60.00%(3/5)
12	100%(2/2)
14	100%(2/2)
17	91.67%(11/12)
21	66.67%(2/3)
28	80.00%(4/5)

### Survival outcomes: Event-Free Survival (EFS) 

The median EFS was 9.6 months (range: 0–39.5 months; 95% CI: 6–16.75). EFS events included relapse (*n *= 6), death from disease progression (*n* = 1), death from pulmonary infection (*n *= 2), treatment-related death (*n *= 3), and MRD conversion to positive after treatment (*n *= 1).

Bridging to Transplant: The median EFS was 10 months for patients who underwent allogeneic hematopoietic stem cell transplantation (*n* = 12), compared to 9 months for the non-transplant group (*n* = 17).

Overall Survival (OS): As of October 2024, the median OS was 29 months (95% CI: 14.9–43.1).

### Correlations between relapse and poor prognostic factors 

Relapse Patterns: Among the 6 patients who relapsed after blinatumomab treatment, 4 (66.7%) experienced bone marrow relapse. Correlation with Genetic Abnormalities: *BCR::ABL1* T315I Mutation: Among 4 patients, 1 (25%) did not achieve MRD negativity, and 2 (50%) experienced early relapses. *E2A::PBX1* Rearrangement: One patient experienced rapid relapse and died within 2 months post-treatment; the other did not achieve remission. BCR::ABL1 P210: Among 4 patients with *BCR::ABL1 P210/ABL*1 ≥ 30%, one died without hematopoietic recovery, two relapsed after treatment, and one achieved CR but maintained persistent fusion gene positivity.

### Treatment-related adverse events 

During treatment, 14 patients (48.3%) experienced Grade 3–4 hematological toxicity. The infection rate was 34.5% (10/29), with 8 cases being Grade 3–4 infections; these included 7 cases of Grade ≥ 3 pulmonary infection. Furthermore, one patient experienced Grade 4 Immune Effector Cell-Associated Neurotoxicity Syndrome (ICANS). This patient recovered after blinatumomab discontinuation and supportive care including dexamethasone, levetiracetam, diazepam, and ICU monitoring, but treatment was consequently interrupted. Another patient developed Grade 2 CRS, which did not require dose adjustment or treatment interruption. One treatment-related death occurred in the entire cohort. Detailed treatment-related adverse events are listed in Table 3.

**Table 3:** Treatment-related adverse events associated with blinatumomab

Adverse Event Category	Patients (*N* = 29)
Any Adverse Event	86.21% (25/29)
Adverse Event Leading to Treatment Discontinuation	3.45% (1/29)
Treatment-Related Mortality	3.45% (1/29)
Grade ≥ 3 Adverse Events	48.28% (14/29)
Hematological Toxicity	
Thrombocytopenia	41.38% (12/29)
Neutropenia	20.69% (6/29)
Anemia	34.48% (10/29)
Infection-Related Events	
Fever	31.03% (9/29)
Pneumonia	21.14% (7/29)
Febrile Neutropenia	10.34% (3/29)
Non-Hematological Toxicity	
Elevated Liver Enzymes	17.24% (5/29)
Hypokalemia	3.45% (1/29)
Diarrhea	6.90% (2/29)
Immune-Related Toxicity	
Cytokine Release Syndrome (CRS)	3.45% (1/29)
Neurological Toxicity (ICANS)	3.45% (1/29)

## Discussion

The treatment of high-risk acute lymphoblastic leukemia (ALL) remains a significant challenge in clinical research. Its complex molecular heterogeneity and drug resistance mechanisms often lead to unsatisfactory durable remission rates with conventional chemotherapy and targeted therapies. This retrospective study, analyzing clinical data from 29 high-risk ALL patients, systematically evaluated the efficacy of blinatumomab and prognostic influencing factors. It reveals the complex relationships between genetic abnormalities, treatment strategies, and survival outcomes, providing important evidence for optimizing individualized therapy for high-risk ALL.

In this study, blinatumomab demonstrated favorable short-term efficacy, with an MRD negativity rate of 82.8%. Before initiating treatment, we assessed CD19 expression on tumor cells in all patients using flow cytometry and administered blinatumomab only to CD19-positive individuals. CD19 loss is known to be associated with molecular mechanisms such as CD19 gene mutations, allelic-specific expression, or low RNA expression. Therefore, it is recommended to incorporate flow cytometric assessment of CD19 expression as a routine screening step prior to blinatumomab initiation to optimize patient selection and improve the likelihood of treatment response [6, 12–14]. Furthermore, all 7 patients with *IKZF1* gene mutations achieved CR and MRD negativity after treatment, whereas patients with *E2A::PBX1* rearrangement responded poorly. This observation suggests that the efficacy of blinatumomab may be associated with the molecular background of the leukemic cells.

The *E2A::PBX1* rearrangement is known to activate the *HOX* gene cluster and is associated with high-risk ALL and poor prognosis [15–20]. In our study, which included only two patients carried this rearrangement, one relapsed rapidly within 2–3 months after achieving MRD negativity, and the other was refractory to treatment. Given the limited sample size of this subgroup (*n* = 2), these observations should be considered strictly hypothesis-generating rather than definitive evidence of intrinsic drug resistance. It is possible that a high burden of *E2A::PBX1*-positive clones (as seen in Patient 23, where the *E2A::PBX1/ABL* ratio increased from 43.24% to 92.16% during treatment) might reduce blinatumomab efficacy, but this requires validation in larger cohorts. Preclinical studies have suggested that impaired T-cell function or E2a/Pbx1-mediated interference with pro-T cell differentiation could contribute to resistance [13, 21, 22]. These factors collectively raise the hypothesis that patients harboring the *E2A::PBX1* rearrangement may have reduced efficacy to blinatumomab, but this requires validation in larger cohorts. Studies suggest that *E2A::PBX1* ALL may be sensitive to the PI3Kδ inhibitor Idelalisib, thus, combining idelalisib with blinatumomab might be a future strategy to explore [23].

The T315I mutation in the *BCR::ABL1* fusion protein confers resistance to all first- and second-generation TKIs [24]. Among our four Ph+ patients with T315I mutations, one did not achieve MRD negativity and two had early relapses. Although the response appeared lower than in non-mutated patients, the small number precludes any statistical comparison. The ALCANTARA study showed a CR rate of 40% with blinatumomab monotherapy in Ph + ALL patients with T315I mutations [25]. The mutation may enhance Abl kinase activity and activate the RAS/ERK pathway, potentially promoting leukemia stem cell survival [26, 27]. Consequently, combining RAS pathway inhibitors could be considered, but this requires further investigation.

In summary, based on our limited sample size, these preliminary observations suggested that in *BCR::ABL1*-positive patients, the presence of TKI-resistant mutations (like T315I) can compromise blinatumomab’s efficacy. In *E2A::PBX1*-rearranged ALL, aberrant HOX gene expression, potentially through upregulation of anti-apoptotic proteins like Bcl-2, may induce immune escape, collectively leading to resistance against blinatumomab [15]. However, these findings are exploratory and require confirmation in larger, well‑controlled studies. Furthermore, in real-world practice, blinatumomab is often used in combination with TKIs, CAR-T, or HSCT; however, consensus is lacking regarding the synergistic mechanisms, optimal sequencing, and toxicity management for such combination strategies.

In this study, blinatumomab was generally well-tolerated. Only one patient discontinued treatment due to ICANS, and one treatment-related death occurred. The primary adverse events were hematological toxicities, with thrombocytopenia (41.4%) and anemia (34.5%) being the most frequent. Approximately 13.8% of patients experienced pulmonary infection or febrile neutropenia. Immune-related toxicities were less common, with the incidence of CRS and ICANS being only 3.4% each. However, transplant-related complications (infections, GVHD) and neurotoxicity still warrant vigilance. For instance, Patient 8 died from cerebral hemorrhage during the transplant conditioning phase, potentially related to immunotherapy-induced endothelial cell activation [28]. Therefore, caution is advised when using high-intensity conditioning regimens in elderly patients or those with pre-existing vascular conditions. Compared to the TOWER clinical trial, our study observed a lower incidence of grade ≥ 3 adverse events (48.28% vs. 70.8%), the incidence of cytokine release syndrome (3.4%) and ICANS (3.4%) was lower than that in TOWER (CRS 4.9%, neurological events 9.4%), possibly due to our routine use of step‑dosing and premedication [6]. Dynamic monitoring of MRD and gene expression (e.g., *BCR::ABL1* quantification) can aid in the early identification of treatment response. For example, among the 4 patients with *BCR::ABL1* P210 ≥ 30%, three experienced Grade 4 hematological adverse events during treatment, one died, and two had short remission durations of only 3 months. Such patients might respond poorly to blinatumomab monotherapy, necessitating alternative combination strategies. Furthermore, our results show an association between longer treatment duration and higher MRD negativity rates, although the considerable variability in treatment duration (7–28 days) and the influence of non-clinical factors (e.g., economic constraints) represent important confounding factors that preclude causal interpretation. One possible mechanism is that blinatumomab’s short half-life leads to rapid clearance from the bloodstream, causing drug levels to fall below effective concentrations prematurely. Shorter treatment durations might also result in insufficient T-cell activation or premature T-cell inactivation, leading to treatment failure [29]. However, these findings should be considered exploratory, prospective studies with standardized treatment protocols are needed to determine the optimal duration of blinatumomab therapy.

Our study provides valuable insights into the clinical outcomes of blinatumomab in high-risk ALL. However, several limitations should be carefully considered. The study population was highly heterogeneous with respect to disease status (newly diagnosed, remission with MRD positivity/negativity, and relapsed/refractory cases) and biological risk factors (Table 1), which limits the interpretability of the observed effects and makes it difficult to fully attribute outcomes to blinatumomab treatment. Due to the relatively small sample size (*n* = 29), we were unable to perform systematic stratified analyses to adjust for this heterogeneity, which also restricts our statistical power within specific genetic subgroups (e.g., only 1 case with *FLT3*-TKD mutation and 2 cases with *TP53*/*DNMT3A* mutations). Further, the results from some subgroups, such as the 100% MRD negativity rate observed in patients with complex karyotypes (6/6) and those aged ≥ 65 years (3/3), appear unexpectedly high compared to the published literature. These observations are likely due to the small sample sizes in these subgroups, which are susceptible to chance variation and may not reflect true biological effects. The statistical analysis was primarily descriptive, formal hypothesis testing was not performed for most subgroup comparisons. Therefore, the observed differences between genetic subgroups (e.g., *E2A::PBX1* vs. others) should be considered hypothesis‑generating rather than confirmatory. To avoid misinterpretation of underpowered tests, we have not reported p‑values for most comparisons. Moreover, a substantial proportion of patients (11/29) were MRD‑negative at baseline, which may introduce selection bias and artificially elevate the overall MRD negativity rate, since the outcome for these patients was maintenance rather than conversion of negativity. Nonetheless, given that this is high-risk ALL, the use of blinatumomab as consolidation therapy is reasonable. Future studies should stratify patients based on baseline MRD status to accurately assess treatment efficacy.

The retrospective design studies inherent selection bias, and the lack of a control group prevents direct comparison of blinatumomab’s efficacy and long-term survival advantage over conventional chemotherapy. Additionally, with only one newly diagnosed patient included, the true efficacy of blinatumomab in treatment-naïve high-risk ALL patients cannot be definitively evaluated. Future multi-center prospective studies, incorporating single-cell sequencing and immune microenvironment analysis, are needed to delve deeper into resistance mechanisms. Moreover, developing a prognostic model for blinatumomab treatment by integrating risk factors such as genetic mutations, MRD dynamics, and imaging features could further enhance the precision of risk stratification. Stratified analyses according to disease status and genetic background should be performed in larger cohorts to provide a clearer understanding of treatment efficacy.

## Conclusion

In summary, blinatumomab represents an important immunotherapy for high-risk ALL, demonstrating a high rate of MRD negativity in this clinically heterogeneous population. Our descriptive observations indicate that its efficacy may be influenced by factors such as *BCR::ABL1* transcript levels, the presence of *BCR::ABL1* T315I mutations, and *E2A::PBX1* rearrangements. Combining blinatumomab with transplantation, targeted therapies remains a potential strategy for optimizing patient outcomes. Future well-controlled trials focusing on individualized treatment approaches guided by molecular subtyping are warranted to overcome current therapeutic limitations and further validate the survival impact.

## Supplementary Information

Supplementary Material 1.

## Data Availability

The datasets used and/or analysed during the current study are available from the corresponding author on reasonable request.

## Abbreviations

ALL: Acute Lymphoblastic Leukemia
B-ALL: B-cell Acute Lymphoblastic Leukemia
BiTE: Bispecific T-cell Engager
MRD: Measurable (Minimal) Residual Disease
CR: Complete Remission
CRi: Complete Remission with Incomplete Hematologic Recovery
CRh: Complete Remission with Partial Hematologic Recovery
MLFS: Morphologic Leukemia-Free State
PR: Partial Remission
NR: No Remission
FCM: Multiparameter Flow cytometry
OS: Overall Survival
EFS: Event-Free Survival
HSCT: Hematopoietic Stem Cell Transplantation
allo-HSCT: Allogeneic Hematopoietic Stem Cell Transplantation
auto-HSCT: Autologous Hematopoietic Stem Cell Transplantation
TKI: Tyrosine Kinase Inhibitor
Ph+: Philadelphia chromosome-positive
Ph-like: Philadelphia chromosome-like
CRS: Cytokine Release Syndrome
ICANS: Immune Effector Cell-Associated Neurotoxicity Syndrome
MICM: Morphology, Immunology, Cytogenetics, Molecular biology
CTCAE: Common Terminology Criteria for Adverse Events
G-CSF: Granulocyte Colony-Stimulating Factor
BBB: Blood-Brain Barrier
LSCs: Leukemia Stem Cells
scFvs: Single-chain Variable Fragments
ICU: Intensive Care Unit
DLBCL: Diffuse Large B-cell Lymphoma
GVHD: Graft-versus-host disease
CAR-T: Chimeric Antigen Receptor T-cell therapy
PCR: Polymerase Chain Reaction
SD: Standard Deviation
IQR: Interquartile Range
